# Gene ontology analysis of expanded porcine blastocysts from gilts fed organic or inorganic selenium combined with pyridoxine

**DOI:** 10.1186/s12864-018-5237-1

**Published:** 2018-11-21

**Authors:** Danyel Bueno Dalto, Stephen Tsoi, Michael K. Dyck, Jean-Jacques Matte

**Affiliations:** 1 Sherbrooke Research and Development Centre, Agriculture and Agri-Food Canada, 2000 College Street, Sherbrooke, QC J1M 0C8 Canada; 2 Department of Agricultural, Food and Nutritional Science, University of Alberta, Edmonton, AB T6G 2P5 Canada

**Keywords:** Amide biosynthesis, Epigenetic, Genomic stability, Peptide trafficking, Pyridoxine, Porcine embryo, Selenium

## Abstract

**Background:**

Gene ontology analysis using the microarray database generated in a previous study by this laboratory was used to further evaluate how maternal dietary supplementation with pyridoxine combined with different sources of selenium (Se) affected global gene expression of expanded porcine blastocysts. Data were generated from 18 gilts randomly assigned to one of three experimental diets (*n* = 6 per treatment): i) basal diet without supplemental Se or pyridoxine (CONT); ii) CONT + 0.3 mg/kg of Na-selenite and 10 mg/kg of HCl-pyridoxine (MSeB_6_10); and iii) CONT + 0.3 mg/kg of Se-enriched yeast and 10 mg/kg of HCl-pyridoxine (OSeB_6_10). All gilts were inseminated at their fifth post-pubertal estrus and euthanized 5 days later for embryo harvesting. Differential gene expression between MSeB_6_10 vs CONT, OSeB_6_10 vs CONT and OSeB_6_10 vs MSeB_6_10 was performed using a porcine embryo-specific microarray.

**Results:**

There were 559, 2458, and 1547 differentially expressed genes for MSeB_6_10 vs CONT, OSeB_6_10 vs CONT and OSeB_6_10 vs MSeB_6_10, respectively. MSeB_6_10 vs CONT stimulated 13 biological processes with a strict effect on RNA binding and translation initiation. OSeB_6_10 vs CONT and OSeB_6_10 vs MSeB_6_10 impacted 188 and 66 biological processes, respectively, with very similar effects on genome stability, ceramide biosynthesis, protein trafficking and epigenetic events. The stimulation of genes related with these processes was confirmed by quantitative real-time RT-PCR.

**Conclusions:**

Gene expression of embryos from OSeB_6_10 supplemented gilts was more impacted than those from MSeB_6_10 supplemented gilts. Whereas maternal OSeB_6_10 supplementation influenced crucial aspects of embryo development, maternal MSeB_6_10 supplementation was restricted to binding activity.

**Electronic supplementary material:**

The online version of this article (10.1186/s12864-018-5237-1) contains supplementary material, which is available to authorized users.

## Background

Although selenium (Se) acts as part of hormones and enzymes influencing the activity of all organs and tissues, the major metabolic role of Se in the body is related to selenoproteins and the antioxidant system. However, in embryos, fetuses, and newborns, the synthesis of selenoproteins from Se-methionine (SeMet) is impaired due to the inactivity of cystathionine gamma lyase in the metabolic pathway, in spite of its mRNA expression [1, 2]. This would imply that, from conception to neonatal age, individuals are not able to convert SeMet into Se-cysteine (SeCys) via the pyridoxine (B_6_)-dependent transsulfuration pathway. In pre-implantation porcine embryos, Dalto et al. [3] showed that, although significant differences in genes expression were observed between embryos from dams supplemented with dietary organic (OSe) vs inorganic Se (MSe), the uterine flushing is a negligible source of Se.

These two crucial aspects suggest that, in pre-implantation embryos, SeMet has an alternative metabolism not related with the antioxidant system and that other sources of Se are transferred from the dam in order to allow selenoprotein synthesis. Because of its undiscriminated metabolism in relation to methionine [4], SeMet is most likely incorporated into general protein, excreted through methylation reactions or recycled after demethylation to Se-homocysteine, generating the Se-derived adenosylmethionine [5]. S-adenosylmethionine (SAM), the sulfur counterpart of Se-adenosylmethionine, is recognized as universal bioactive methyl donor donating its methyl group to a large number of methyl acceptors [6, 7] with profound impacts on DNA synthesis, DNA protection and repair, cellular metabolism, and cell proliferation. Consequently, it can generate direct effects on embryo/fetal growth [8, 9] and epigenetic modifications [10]. Regarding the transfer of Se from dam to pre-implantation embryos, to the best of our knowledge, this aspect of SeMet metabolism is not known in porcine embryos developing under such specific uterus conditions. In a previous study using a unique microarray platform, Dalto et al. [3] have evaluated genes expression of porcine expanded blastocysts (PEB) from gilts fed organic or inorganic Se and B_6_. In that report, the focus was put on aspects related with Se metabolism and antioxidation, using a very restrictive gene selection approach. Therefore, potential global metabolic effects of Se on PEB were not addressed.

Considering the uniqueness of these samples (embryos collected at day 5 of gestation from gilts under specific dietary treatments and controlled experimental conditions), complementary analyses were performed on samples and on the dataset from the previous study. In the present report, a gene ontology approach was applied to provide a global overview of the effects of Se in PEB and, considering the lack of information on that matter, these new findings allowed proposing hypotheses in relation to Se metabolism at this stage of development.

## Material and methods

Most aspects of the Materials and Methods for the present study have been previously described in Dalto et al. [3]. Therefore, the present description summarizes only the relevant information needed for the present objectives.

Eighteen Yorkshire-Landrace gilts were selected at 96.1 ± 4.6 kg of body weight and 135–170 days of age. For at least 14 days, they were fed ad libitum a basal breeding/gestation diet without Se and B_6_ supplements but in excess of the recommended NRC [11] requirements for all other ingredients. Estrus detection was initially performed once daily, but was increased to twice daily for the detection of the fifth post-pubertal estrus. From the onset of the first post-pubertal estrus, gilts were placed in individual stalls, daily feed allowance was limited to 2.8 kg, and they were randomly assigned (according to their body weight and blood concentration of Se) to one of the three experimental diets (*n* = 6 per treatment): i) basal diet containing 0.3 and 2.4 mg/kg of native Se and pyridoxine, respectively (CONT); ii) basal diet + 0.3 and 10 mg/kg of supplemental inorganic Se (Na-selenite) and HCl-pyridoxine, respectively (MSeB_6_10); and iii) basal diet + 0.3 and 10 mg/kg of supplemental organic Se (Se-enriched yeast) and HCl-pyridoxine, respectively (OSeB_6_10). At the fifth post-pubetal estrus, all gilts were inseminated with pooled semen from the same three Duroc boars. When estrus was detected in the morning, gilts were inseminated 8 and 24 h later. When estrus was detected in the afternoon, inseminations were performed 16 and 24 h later. All gilts were euthanized 5 days after the first insemination. The euthanasia procedure involved the sedation of the animal (Stresnil 2.5 ml/kg; Vetoquinol) followed by stunning using a penetrating captive bolt (TED; Bock Industries) and bleeding. Average body weight was 138.5 ± 6.3 and 181.3 ± 6.3 kg at the initiation of treatment and at the end of the experiment, respectively.

All procedures related with embryo (day 5 of gestation) collection and storage, total RNA extraction, microarray processes, and bioinformatics tools were previously described by Dalto et al. [3]. A two-color microarray with a dye-swap replicate was performed for the MSeB_6_10 vs CONT and OSeB_6_10 vs CONT comparisons and a reference design [12] was chosen using the same CONT group used in the previous comparisons as a reference for a reliable indirect comparison of gene expression for OSeB_6_10 vs MSeB_6_10. Annotation of all the unknown genes was performed using porcine Sscrofa10.2 database (accession GCF_000003025.5). The list of 14,536 unique gene symbols (GSs) from re-annotated EMPV1 used as background for Gene Ontology (GO) Enrichment Analysis is shown in the Additional file 1: Table S1. GORILLA Classification System (http://cbl-gorilla.cs.technion.ac.il/) was used for the GO analysis with two unranked lists of genes (target and background lists). These results were further uploaded to REVIGO (http://revigo.irb.hr/) to provide a summarized view of results.

### Quantitative real-time RT-PCR (RT-qPCR) and analysis

A two-step quantitative real-time RT-PCR (RT-qPCR) was performed on the same aRNA samples from Dalto et al. [3]. Based on the microarray data, two genes related with epigenetic events: histone acetyltransferase 1 (*HAT1*) and histone deacetylase 1 (*HDAC1*); two genes related with establishment of protein localization to endoplasmic reticulum: signal peptidase complex subunit 1 (*SPCS1*) and signal recognition particle 9 (*SRP9*); two genes related with protein catabolism: anaphase promoting complex subunit 1 (*ANAPC1*) and cullin 1 (*CUL1*); one gene related with transcription initiation and DNA repair: cyclin dependent kinase 7 (*CDK7*); and one gene related with the nucleotide excision repair system: growth arrest and DNA-damage-inducible gamma interacting protein 1 (*GADD45GIP1*) were chosen for RT-qPCR validation using the methodology described by Dalto et al. [3]. The reference gene that was found to be the least affected by treatments and, therefore, used for normalization was peptidylprolyl isomerase A (*PPIA*). The sequence information of the primers is given in Table 1.

**Table 1:** Primer sequences used for RT-qPCR amplifications of reference gene and selected genes in porcine expanded blastocysts

Genes	Primer sequences (5′ → 3′)	GenBank Accession no.	Product size (bp)	Amplification efficiency (%)
CDK7	(F) TGGAATCCCGCTACAACATATC	XM_021076538.1	111	0.997
	(R) AATGCCTGTGTGGCTGTAA			
SPCS1	(F) TGGATTACAAGGGCCAGAAG	NM_001114288.2	91	0.996
	(R) GCCACGTACCCGTAGATAAAT			
GADD45GIP1	(F) ATTGAAGAGTGCATGGCTAAGA	XM_003123339.3	89	0.999
	(R) TTGTCTGCTTGCTCCTTCTC			
ANAPC1	(F) CCTGTTTCCTTGTCTACCACTC	XM_013995810.1	118	0.999
	(R) ACTGGCATCTTGAGCTGTTTA			
HDAC1	(F) GGGATTGATGACGAGTCCTATG	XM_013999116.2	104	0.999
	(R) GAGTCAGAGCCACACTGTAAG			
CUL1	(F) CAGTTACTCGGAGAAGTCCTAAC	XM_013979868.1	117	0.999
	(R) ACCATCAACTCGCTCCAAATA			
SRP9	(F) CCTGCCCAATTCTCCCTTTAT	XM_003130540.6	90	0.994
	(R) CAATCCCATACTTCCGGTTTACT			
HAT1	(F) GCAGTAGAGGCTCAACAGAAG	XM_003483674.4	91	0.999
	(R) CACTCATGTCAGTTACCAGTAGTC			

For RT-qPCR analysis, the Relative Expression Software Tool 2009 (REST; http://rest.gene-quantification.info/) was used to implement a randomized test [13] and to assess statistical significance of the up- or down-regulation of the target genes after normalization to the reference gene. Statistical analyses were considered significant at *P* ≤ 0.05.

## Results

### Differentially genes expression profiles

The direct comparison of MSeB_6_10 vs CONT embryos showed a total of 559 genes in the PEB, with 293 up-regulated (52.4%) and 266 down-regulated (47.6%) in MSeB_6_10. For the OSeB_6_10 vs CONT comparison, a total of 2458 differentially expressed genes were found, with 1658 up-regulated (67.5%) and 800 down-regulated genes (32.5%) in OSeB_6_10. Regarding the reference design comparison (OSeB_6_10 vs MSeB_6_10), there were 1547 genes from which OSeB_6_10 had 1096 up-regulated (70.8%) and 451 down-regulated genes (29.2%) compared with MSeB_6_10. Detailed information on the above comparison between treatments (EMPV1 probe ID, log2 fold change, *P*-value, and gene symbol) are given in Additional file 2: Table S2.

### Gene ontology enrichment analyses

Regarding the MSeB_6_10 vs CONT comparison, 6 distinct cellular components, 1 molecular function, and 13 biological processes were stimulated (*P* ≤ 8.94 × 10^− 8^; FDR q-value ≤9.57 × 10^− 5^) according to GORILLA, which were respectively included in 2, 1 and 5 GO terms on REVIGO (log_10_ *P* ≤ − 7.05). The summarized description of REVIGO shows that MSeB_6_10 stimulated cellular components of the cytosol, more specifically intracellular organelle parts and the macromolecular complex, functioning as a RNA binding element. The metabolic role of MSeB_6_10 in PEB is related with cellular metabolism, especially nucleobase-containing compound metabolic process. Although not categorized as cellular metabolism, other GO term identified was translational initiation. By analyzing each individual gene of the MSeB_6_10 x CONT dataset, few genes related with epigenetic events were found [*HDAC1*, *HDAC9*, lysine demethylase 5C (*KDM5C*) and 5 methyltransferases].

For the comparison of OSeB_6_10 vs CONT, 47, 20, and 118 cellular components, molecular functions, and biological processes were respectively stimulated (*P* ≤ 8.30 × 10^− 8^; FDR q-value ≤1.15 × 10^− 5^) according to GORILLA, which were included in 5, 12, 28 GO terms on REVIGO respectively (log_10_ *P* ≤ − 7.01). The summarized description of REVIGO shows that OSeB_6_10 stimulated cellular components of the nucleus and catalytic complex. A more detailed observation reveals that OSeB_6_10 is widely distributed within the cell, being a component of the nucleus (spliceosome complex), mitochondria (matrix and inner membrane protein complex), and ribosomes as well as ribonucleoprotein complex (related with both ribosome and spliceosomal complex), and oxidoreductase complex. According to REVIGO, the functions of OSeB_6_10 are mainly related with protein binding and translation initiation factor activity. Among these two metabolic functions the most representative GO terms were protein binding and translation factor activity - RNA binding. Although not related with those two main metabolic functions, other interesting GO terms were NADH dehydrogenase (ubiquinone) activity, structural constituent of ribosome, and ribonucleoprotein complex binding. Among the 28 biological processes on REVIGO for OSeB_6_10 x CONT, results show that RNA processing, establishment of protein localization to organelle, and macromolecular complex subunit organization are the most relevant. Among these processes, GO terms related with DNA repair, mRNA processing, mRNA translation, intracellular ceramides synthesis, and intracellular peptides trafficking stood out (Additional file 3: Table S3). By analyzing each individual gene of the OSeB_6_10 x CONT dataset, many genes related with epigenetic events were found, among them the most relevant are methyl-CpG binding domain proteins (*MBD3*, *MBD4* and *MBD3L5*), *HAT1*, *HDAC1*, *HDAC9*, *KDM5C*, protein arginine methyltransferases (*PRMT7* and *PRMT10*), DNA methyltransferases (*DNMT1* and *DNMT3B*), and 17 other methyltransferases.

The indirect comparison of Se sources (OSeB_6_10 vs MSeB_6_10) showed that 31 cellular components, 10 molecular functions, and 66 biological processes were stimulated (*P* ≤ 9.41 × 10^− 8^; FDR q-value ≤3.36 × 10^− 5^) according to GORILLA and were respectively represented mainly by 4, 5, 9 GO terms on REVIGO (log_10_ *P* ≤ − 7.34). The summarized description of REVIGO shows a stimulus in cellular components of the nucleus and catalytic complex. A more detailed observation reveals that Se is widely distributed within the cell, being a component of the nucleus, mitochondria (inner membrane protein complex), and ribosomes. In terms of molecular functions, Se acts mainly in translation factor activity (related with RNA binding). Besides that, Se also acts on protein binding and as a structural constituent of ribosome. Among all biological processes, REVIGO results show that RNA processing and protein localization to endoplasmic reticulum are the most relevant (Additional file 3: Table S3). By analyzing each individual gene of the OSeB_6_10 x MseB_6_10 dataset, many genes related with epigenetic events were found, most of them were similar to those found on OSeB_6_10 x CONT. In this sense, out of the GORILLA GO terms found in OSeB_6_10 vs MSeB_6_10 for cellular components, molecular functions, and biological processes, 30 (96.8%), 10 (100.0%), and 64 (97.0%), respectively, were also found in the OSeB_6_10 vs CONT comparison, indicating that most of the effects observed on OSeB_6_10 vs MSeB_6_10 came from OSe.

The complete list of GO terms related with each comparison is shown in Additional file 3: Table S3.

### Validation of microarray data by RT-qPCR

**Fig. 1:**
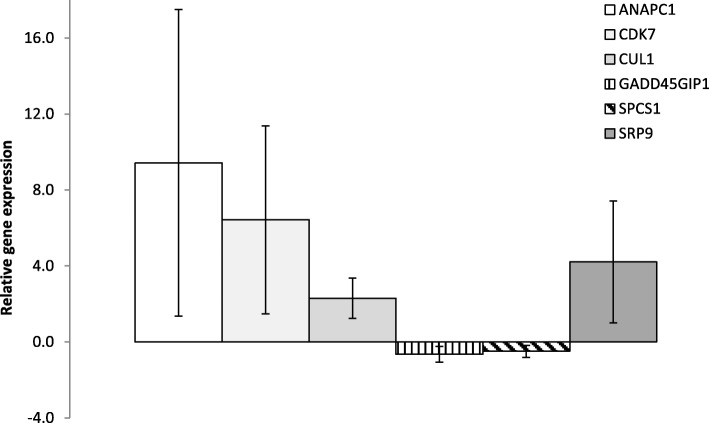
RT-qPCR expression trends for *ANAPC1*, *CDK7*, *CUL1*, *GADD45GIP1*, *SPCS1*, and *SRP9* in porcine expanded blastocysts recovered from OSeB_6_10 supplemented gilts, shown as relative gene expression to CONT (± SEM). *PPIA* was used to normalize the mRNA expression levels. OSeB_6_10 = basal diet supplemented with 0.3 mg/kg of Se-enriched yeast and 10 mg/kg of hydro-chloride pyridoxine

Treatment effects on the expression of genes related with the main GO terms were validated by RT-qPCR. The expression of genes *CUL1*, *ANAPC1*, *SRP9* and *CDK7* was up-regulated whereas *GADD45GIP1* and *SPCS1* were down-regulated in the comparison OSeB_6_10 vs CONT (Fig. 1). For OSeB_6_10 vs MSeB_6_10, RT-qPCR analysis indicated no difference in relative *HAT1* expression whereas *HDAC1* was up-regulated by a mean factor of 1.88. Selected genes from MSeB_6_10 vs CONT comparison did not provide reliable results and were not considered in the present study.

## Discussion

The present study describes a global metabolic perspective of the effects of different Se sources associated with B_6_ on PEB after maternal supplementation, and generates some hypothesis based on these new findings. Although the same microarray database was used by Dalto et al. [3] and the present study, those authors used the pig genome map draft 9.0 for genes annotation and a more restrictive statistical approach in order to evaluate highly affected processes, functions, and components related with each treatment. In the present study re-annotation was made using the updated pig genome map version 10.2 and parameters related with statistics and genes selection were chosen in order to provide a global overview of the effects of Se in PEB.

Considering that the present results were observed in self-regulated organisms (expanded blastocysts) that are genetically dissimilar (half allogenic) from those directly receiving the treatments (dams), such effects would require major metabolic alterations. In fact, Dalto et al. (2015; original database) reported that maternal blood Se was clearly modulated by dietary Se. Therefore, non-Se fractions of dietary OSe (mainly yeast) or MSe (Na + ion) are unlikely involved in such major systemic metabolic changes.

Both sources of Se (MSe and OSe) are used as dietary supplements for pigs but, depending on the Se status of the animal, they follow different metabolic pathways [4] before producing a common metabolite (selenide) required for the synthesis of selenoenzymes [5]. According to Burk et al. [14], in mouse, MSe does not take part of the maternal methionine pool but is mainly incorporated in selenoproteins whereas OSe is mostly deposited in the methionine pool with limited synthesis of selenoproteins. Considering that the uterine flushing of 5 days gestation gilts is a negligible route of Se transfer for the embryo [3], the Se content of pre-ovulatory oocytes is likely to be the main source of this mineral for pre-implantation porcine embryos. Therefore, whatever the source of maternal dietary Se, the embryo Se content would derive from an organic metabolite, either the oocyte SeMet pool and/or oocyte selenoproteins.

In this sense, we hypothesize that for both MSe and OSe supplemented gilts, maternal (oocyte) selenoproteins serve, after proteolysis, as a common source of SeCys for either embryo protein deposition or selenoproteins synthesis, whereas oocyte SeMet (OSe) might be directly deposited into embryo proteins and/or undergo transmethylation, because the transsulfuration pathway is not fully functional in embryos [1].

This hypothesis involving a common source of embryonic Se between OSe and MSe is indirectly supported by the present and previous studies. In fact, all MSeB_6_10 vs CONT GO terms were also found in OSeB_6_10 vs CONT comparison, suggesting that both sources of Se share a common metabolism but also that OSe may have additional metabolic pathways. Fortier et al. [15] and Dalto et al. [16] reported that even after placentation (30 days of gestation) porcine embryos from MSe or OSe supplemented gilts have similar Se-dependent glutathione peroxidase (Se-GPX) activities, indicating that independently of maternal Se source, embryos may obtain their Se content from a common intermediary Se metabolite, which is subjected to similar regulations for the synthesis of Se-GPX. Nevertheless, in both studies, the Se content of embryo from OSe supplemented gilts was greater than in those from MSe supplemented gilts, indicating the presence of different and/or additional Se metabolic pathways in OSe embryos.

### DNA repair

DNA damage triggers repair pathways, but also signalling pathways that stimulate cell cycle checkpoints, apoptosis, transcription, and chromatin remodelling [17]. It has been proposed that, among the mechanisms for monitoring DNA integrity, cells may detect stalled RNA polymerases or abortive transcripts, activating DNA damage signaling [18]. The present results for OSeB_6_10 x MSeB_6_10 indicate that Se acts in one of these signaling pathways, the nucleotide excision repair (NER). More specifically, it affects the transcription-coupled NER (TC-NER), a specialized sub-pathway of NER responsible to repair DNA lesions in transcribed strands and coupled to active transcription [19, 20]. The high similarity between GO terms for OSeB_6_10 vs CONT and OSeB_6_10 vs MSeB_6_10 and the up-regulation of *CDK7* (gene that links transcription initiation, DNA repair, and cell cycle) in OSeB_6_10 vs CONT suggests a major influence of OSe in this process.

SeMet (main OSe form) have been reported to impact protein p53 activity [21], a protein that under conditions of permanent blockage of transcription may stimulate p53-dependent apoptosis [22]. Therefore, the fast removal of transcription blocking lesions is crucial to avoid the detrimental effects of transcription inhibition. Studies have proposed a direct role of p53 in the OSe-induced activation of the NER pathway through its interaction with NER-associated proteins [23–25]. However, in the present data, none of the genes proposed to link p53 and NER [growth arrest and DNA damage inducible alpha (*GADD45A*), proliferating cell nuclear antigen (*PCNA*) and apurinic/apyrimidinic endodeoxyribonuclease 1(*APE1*)] [26] were expressed. Moreover, *GADD45GIP1*, a gene induced by p53 and that inhibits G1 to S phase of cell cycle progression was, in fact, down-regulated. Therefore, the present study suggests the possible role of alternative mechanisms for OSe in maintaining genomic stability in PEB through TC-NER. The present data suggests two likely possibilities, the activation of caspases by C-terminal-modified *TXNR* or ubiquitylation of damage-arrested RNA polymerase.

C-terminal-modified *TXNR* is unable to reduce TXN, but may replace the p53 apoptotic function via activation of caspases [27]. Interesting, Dalto et al. [3] reported a down-regulation of *TXN* in OSeB_6_10 x CONT and OSeB_6_10 x MSeB_6_10, whereas in the present study *CASP3*, *6*, and *7* were up-regulated in these comparisons. For damage-arrested RNA polymerase, it might be released from the template by a mechanism that leads to its ubiquitylation and degradation [22], in which genes ERCC excision repair (ERCC) and cullin (CUL) play a main role. In OSeB_6_10 x CONT, genes encoding proteins that are important in ubiquitination of proteins involved in cell cycle progression, signal transduction and transcription were up-regulated [*ANAPC1*, *CUL1*, *CUL2*, DDB1 and CUL4 associated factor 7 (*DCAF7*) and *ERCC8*]. Globally, these results on DNA repair are consistent with the numerically greater amount of viable embryos collected from OSeB_6_10 gilts by Dalto et al. [3], and this, in absence of degenerated embryos.

### mRNA processing

After the formation of the precursor mRNa (pre-mRNA) in the nucleus, it has to be converted to mature mRNA by splicing [28] in order to be translated. Pre-mRNA splicing occurs in the spliceosome, a large ribonucleoprotein complex composed of five U-type small nuclear ribonucleoprotein particles (snRNPs) and splicing factors [29].

The present data for OSeB_6_10 x CONT indicates that OSe acts on the ribonucleoprotein complex assembly. Although SeMet may have a structural function by replacing methionine in proteins, in PEB the most important effect of OSe appears to be on splicing factors activity. In fact, many dead-box proteins, pre-mRNA processing factors, snRNPs polypeptides, and other splicing factors (among them serine/arginine-rich splicing factors, poly (A) polymerases, and cleavage stimulation factors 3′ pre RNA) were impacted by maternal OSe supplementation.

During the second catalytic step of splicing, the exon junction complex (EJC) is formed on the pre-mRNA strand at the exon-exon junction [30, 31]. In the present data for OSeB_6_10 x CONT, two [eukaryotic translation initiation factor 4A3 (*EIF4A3*) and mago homolog, exon junction complex core component (*MAGOH*)] of the three core proteins of the EJC were expressed. This protein complex has major influences on translation, localization of the spliced mRNA, and mRNA surveillance. According to Brogna & Wen [32], in mammalian cells, EJC mediates the link between splicing and nonsense-mediated decay pathway (NMD), a surveillance translation-coupled mechanism that eliminates mRNAs containing premature translation-termination codons [33].

The NMD pathway is assumed to affect selenoproteins expression because they contain multiple SeCys residues, which are encoded by the UGA codon that normally signals translation termination. In selenoproteins, however, SeCys insertion sequence (SECIS) recognizes UGA as a SeCys codon rather than as a stop signal. Under Se deficiency, Seyedali & Berry [34] showed that NMD may act on selenoproteins mRNA. In the present data, SECIS binding protein 2 (*SECISBP2*) was up-regulated in OSeB_6_10 x MSeB_6_10, suggesting that selenoproteins synthesis was active. In fact, selenoprotein K was up-regulated in both OSeB_6_10 x CONT and OSeB_6_10 x MSeB_6_10. For the most known selenoproteins (glutathione peroxidases, iodothyronine deiodinases, and thioredoxin reductases), none of them was differentially expressed. This, however, does not imply that their synthesis was impaired but that maternal Se supplementation did not further stimulate their synthesis compared to the control diet or between Se sources.

### mRNA translation

The present data indicates that besides the function of OSe as a structural component of the ribosome, both MSe and OSe play roles in translation, stimulating RNA binding factors and influencing translational initiation. A deeper evaluation of individual genes in these GO terms revealed that, under the present experimental conditions, Se is likely to influence specifically the Cap-dependent initiation. This process involves the interaction of the eukaryotic translation initiation factor (eIF) complex and the 5′ cap as well as with the 5′ untranslated region [35]. Additionally, the transport of the initiator tRNA, which encodes the amino acid methionine in eukaryotes, to the P-site of the small ribosomal subunit is performed by eIF2. This protein is also responsible for signaling the dissociation of several factors from the small ribosomal subunit, among them eIF3 that avoids the premature binding of the large ribosomal subunit, leading to the association of the large subunit and translation elongation [36]. Except for eIF5 and eIF6 that were not expressed by MSeB_6_10 x CONT, all three comparisons have expressed many subunits of important eIFs.

### Intracellular ceramides synthesis

mRNA translation was expectedly related with peptide biosynthesis, which in turn was related with amide biosynthesis. Although the association between amides and peptides may be due to the presence of amide bonds in the forming protein, a deeper evaluation of individual genes revealed that the GO term amide biosynthetic process had many genes related with two out of the three pathways of ceramides biosynthesis.

The de novo pathway begins with the formation of 3-keto-dihydro-sphingosine from palmitoyl-CoA and serine by serine palmitoyl-transferase (*SPTLC2*) in the endoplasmic reticulum. Further, 3-keto-dihydro-sphingosine is reduced by 3-keto-dihydro-sphingosine reductase (*KDSR*) to form dihydro-sphingosine followed by an acylation by ceramide synthase (*CERS1*, *CERS5*, and *CERS6*) to form dihydro-ceramide, which is desaturated by delta 4-desaturase (*DEGS*) to form ceramide. Ceramide is subsequently transported to the Golgi apparatus where it is further metabolized and the outcomes transported to the plasma membrane [37].

Breakdown pathways allow the reversion of sphingolipids back to ceramide. One of the most biologically important reactions is the breakdown of sphingomyelin in the cell membrane releasing ceramide. Considering that sphingomyelin is the most abundant complex sphingolipid in human cells, its coordinated breakdown is an essential part of membrane homeostasis [38]. Besides the breakdown of sphingosine by ceramide synthases, the present results support that OSe plays a role in the breakdown of sphingomyelin by sphingomyelin phosphodiesterase (*SMPD4* and *SMPDL3A*) and in the synthesis of sphingosine by N-acylsphingosine amidohydrolase 1 (*ASAH1*) and/or alkaline ceramidase 3 (*ACER3*). Sphingosine may enter the cell or it is metabolized by sphingosine kinase 1 (*SPHK1*), a protein regulated by SPHK1-interacting protein (*SPHKAP*), to form sphingosine-1-phosphate. The latter may be dephosphorylated by phospholipid phosphatase 1 (*PPAP2A*) to resynthesize sphingosine. In the cell, sphingosine can either go directly to the endoplasmic reticulum or enter the mitochondria where it will be metabolized back to ceramide and released into the endoplasmic reticulum [37]. Among all genes mentioned in de novo and breakdown pathways, only *KDSR* and *SPHK1* were not expressed in OSeB_6_10 x CONT and/or OSeB_6_10 x MSeB_6_10.

Simple sphingolipids have significant signaling and regulatory roles within cells, with serious consequences for mammalian physiology [37]. Ceramide and sphingosine-1-phosphate have been shown to be important mediators in the signaling cascades involved in apoptosis, proliferation, differentiation, cell growth arrest, inflammation, cell migration and adhesion. In this sense, many factors known to promote the synthesis of sphingosine-1-phosphate [39] were expressed in the present data [platelet-derived growth factor subunit A (*PDGFRA*), insulin-like growth factor 1 receptor (*IGF1R*), vascular endothelial growth factor β (*VEGFB*), tumor necrosis factor-related genes (*TNFRSF21*, *TNFAIP8L3*, *TNFSF4*, and *TNFRSF9*), and low-density lipoprotein receptors (*LRP2* and *LRP6*). Globally, these results are once again consistent with the numerically greater amount of advanced-stage embryos collected from OSeB_6_10 gilts by Dalto et al. [3].

### Intracellular peptides trafficking

Besides the effects on peptides and lipid-related metabolites biosynthesis, OSeB_6_10 x CONT and OSeB_6_10 x MSeB_6_10 comparisons also showed an impact on their transport via the signal recognition particle (SRP)-dependent cotranslational pathway of protein targeting to membrane. The cotranslational pathway uses SRP to deliver secretory proteins to a membrane-bound protein-conducting channel (translocon), which is present in the endoplasmic reticulum membrane, concomitantly with their synthesis in ribosomes [40]. In mammals, SRP consists of six proteins (encoded by *SRP9*, *SRP14*, *SRP19*, *SRP54*, *SRP68* and *SRP72*) and a 7S RNA, most of which had genes expressed in the above mentioned comparisons. This process begins with the recognition of the signal peptide of the protein by SRP during the protein synthesis in the ribosome [41], with further insertion of the nascent protein into the translocon [42]. The translocon complex consists of oligosaccharyl transferase complex, the translocon-associated protein (TRAP) complex, and the translocating chain-associating membrane protein (TRAM), besides the central element Sec61 [43]. For the present data, many genes related with the translocon complex (3 *SEC61* subunits, *STT3A*, *STT3B*, and 7 *TRAPPC* subunits) were expressed in OSeB_6_10 x MSeB_6_10 and/or OSeB_6_10 x CONT. Once the nascent polypeptide has been translocated into the endoplasmic reticulum membrane, the signal sequence is cleaved by signal peptidases (GTP hydrolysis) [44, 45], some of which were expressed (*SPCS1*, *SPCS2*, and *SPCS3*) in OSeB_6_10 x MSeB_6_10 and/or OSeB_6_10 x CONT.

Signal peptidases are also found in the mitochondria import machinery [46]. The pre-protein containing peptide signals targeting the mitochondria is bound by translocases of outer membrane (TOM) and transported through the intermembrane space by translocases of inner membrane (TIM) [47]. Among the three mitochondrial TOMs, those responsible for binding pre-sequences and internal targeting peptides (*TOM20* and *TOM22*) were expressed in OSeB_6_10 x MSeB_6_10 and OSeB_6_10 x CONT. Additionally, *TIM23*, which acts as a translocator of pre-proteins for the mitochondrial matrix, the inner membrane, and the intermembrane space, was affected in these same comparisons.

Exclusively for the comparison OSeB_6_10 x CONT, the present data show an effect on mitochondrial respiratory chain complex assembly, more specifically the NADH dehydrogenase (ubiquinone) activity (21 NADH dehydrogenase and 5 cytochrome c genes stimulated). The evaluation of individual genes in these GO terms showed that Se acts mainly as a structural component, contrary to its expected antioxidant action against reactive oxygen species in the electron transport chain.

### Epigenetics

It is already known that maternal diet may play a crucial role in epigenetic programming of conceptus development [48] and that methyl dietary supplements (such as SeMet) can alter the methylation of specific imprinted genes [49]. For this reason, an individual search for epigenetic-related genes was performed. Many important genes related with epigenetic events were expressed in OSeB_6_10 x MSeB_6_10 and/or OSeB_6_10 x CONT (*DNMT1*, *DNMT3B* and 19 other methyltransferases as well as *MBD3*, *MBD4*, *MBD3L5*, *HAT1*, *HDAC1*, *HDAC9*, *KDM5C*, and WDR5) whereas only a few in MSeB_6_10 x CONT (*HDAC1*, *HDAC9*, *KDM5C*, and 6 methyltransferases).

Epigenetic processes are dynamic during embryogenesis partially because of the significant amount of DNA synthesis that occurs in this period [50]. During the pre-implantation period, embryos’ epigenome is particularly susceptible to environmentally induced modifications. At this period, de/methylation of DNA and histones modification occurs. Effects of supplemental Se on global and gene-specific DNA methylation have been reported [51, 52], but never in PEB after maternal Se supplementation. Transfer of methyl groups from SAM to the 5-carbon position of cytosine by DNA methyltransferases (*DNMTs)*, results in 5-methylcytosine (5 mC) [53]. In contrast, DNA demethylation is not catalyzed directly but results from either DNA replication-coupled dilution or replacement of 5 mC. For histones, the interference of nutrients occurs mainly through modulation of histone modifying enzymes and via interference with substrate availability. According to Narayan et al. [54], selenoprotein biosynthesis is crucial for selenite-induced modulation of histone H4 acetylation, supporting the conclusion of Dalto et al. [3] that, although not influenced by Se sources with B_6_, PEB are potentially capable of synthesizing selenoproteins.

A tempting interpretation for the higher impact of OSe (SeMet) compared to MSe (selenite) on epigenetic events would be to link it to the synthesis of SAM during the demethylation of SeMet to Se-homocysteine. However, as the metabolic ratio between methionine and SeMet is generally considered to be above 3000/1 [55], it appears likely that the contribution of SeMet to SAM (a sulfurized metabolite) synthesis is negligible. Nevertheless, this is dependent upon the specific biosynthetic substitution of methionine by SeMet in PEB cells which has never been determined in pigs. Using recombinant human annexin V expressed in *E. coli* as a model, Budisa et al. [56] showed that, independently of experimental SeMet concentration (ranged from 0.3 to 0.8 mM), methionine was fully replace by SeMet. However, it has to be stated that the biosynthetic substitution of methionine by SeMet may vary considerably between species.

If the above mentioned ratio of 3000/1 between methionine and SeMet also applies to PEB, it has to be assumed that it is reflected on intermediary metabolites of the transmethylation pathway, such as Se-homocysteine. Therefore, the demethylation of SeMet to Se-homocysteine would also be quantitatively negligible within the total (sulfur + Se) homocysteine levels. However, some studies showed an inverse correlation between Se status and total (sulfur + Se) homocysteine levels [57, 58] indicating the important impact of Se on intermediary metabolites of the transmethylation pathway. Consequently, the question remains as whether selenized molecules would be more active than sulfurized ones within the transmethylation pathway or whether alternative Se-dependent metabolic pathways would be present in PEB to influence epigenetic events.

## Conclusions

Global gene expression of PEB from OSe supplemented gilts was more impacted than those of MSe supplemented gilts. Maternal OSe supplementation influenced PEB genomic stability, intracellular ceramides synthesis and peptides trafficking, whereas maternal MSe supplementation was restricted to RNA binding. Different concentrations of Se metabolites (SeMet and SeCys) in PEB could explain these great differences based on their distinctive metabolic pathways and functions. Although the present study allows only inferences in this regard, these results are consistent with the better morphological and physiological development of embryos from OSeB_6_10 supplemented gilts as previously reported by this laboratory [3].

Considering the particular transfer of Se from gilts to PEB, the immature transsulfuration pathway generating two metabolic pathways for Se (one for SeMet and other for SeCys), and the possible existence of alternative pathways for epigenetic control, the impact of maternal Se supplementation on transmethylation and transsulfuration pathways in PEB deserves to be further explored.

## Additional files

Additional file 1: **Table S1.** List of unique genes symbols from re-annotated EMPV1 used as background for Gene Ontology Enrichment Analysis. (XLSX 817 kb)

Additional file 2: **Table S2.** Complete list of expressed genes related with MSeB_6_10 vs CONT, OSeB_6_10 vs CONT, and OSeB_6_10 vs MSeB_6_10 comparisons. (XLSX 508 kb)

Additional file 3: **Table S3.** Description of data: Complete list of Gene Ontology terms (GORILLA and REVIGO) related with MSeB_6_10 vs CONT, OSeB_6_10 vs CONT, and OSeB_6_10 vs MSeB_6_10 comparisons. (XLSX 1678 kb)

## Abbreviations

B_6_: Vitamin B_6_
CONT: Control treatment
EJC: Exon junction complex
GO: Gene ontology
GS: Gene symbol
MSe: Mineral selenium
MSeB_6_10: Basal diet + 0.3 and 10 mg/kg of supplemental inorganic Se and HCl-pyridoxine
NER: Nucleotide excision repair
NMD: Nonsense-mediated decay
OSe: Organic selenium
OSeB_6_10: Basal diet + 0.3 and 10 mg/kg of supplemental organic Se and HCl-pyridoxine
PEB: Porcine expanded blastocyst
pre-mRNA: Precursor mRNa
RT-qPCR: Quantitative real-time RT-PCR
SAM: S-adenosyl methionine
Se: Selenium
SECIS: Selenocysteine insertion sequence
SeCys: Selenocysteine
Se-GPX: Selenium-dependent glutathione peroxidase
SeMet: Selenomethionine
snRNP: Small nuclear ribonucleoprotein particle
SRP: Signal recognition particle
TC-NER: Transcription-coupled nucleotide excision repair
TIM: Translocases of inner membrane
TOM: Translocases of outer membrane
TRAM: Translocating chain-associating membrane protein
TRAP: Translocon-associated protein
